# Novel Monoclonal Antibodies and Recombined Antibodies Against Variant SARS-CoV-2

**DOI:** 10.3389/fimmu.2021.715464

**Published:** 2021-08-30

**Authors:** Jiajia Xie, Chengchao Ding, Jun He, Yuqing Zhang, Shuangshuang Ni, Xiangyu Zhang, Qingqing Chen, Jing Wang, Lina Huang, Hongliang He, Wenting Li, Huan Ma, Tengchuan Jin, Siping Zhang, Yong Gao

**Affiliations:** ^1^The First Affiliated Hospital of USTC, Division of Life Sciences and Medicine, University of Science and Technology of China, Hefei, China; ^2^Department of Microbiology, Anhui Provincial Center for Disease Control and Prevention, Hefei, China; ^3^Division of Life Sciences and Medicine, Laboratory of Structural Immunology, University of Science and Technology of China (USTC), Hefei, China

**Keywords:** SARS-CoV-2, neutralizing antibody, recombined antibody, mutation resistance, RBD binding affinity

## Abstract

The mutants resulted from the ongoing SARS-CoV-2 epidemic have showed resistance to antibody neutralization and vaccine-induced immune response. The present study isolated and identified two novel SARS-CoV-2 neutralizing antibodies (nAbs) from convalescent COVID-19 patients. These two nAbs (XG81 and XG83) were then systemically compared with nine nAbs that were reconstructed by using published data, and revealed that, even though these two nAbs shared targeting epitopes on spike protein, they were different from any of the nine nAbs. Compared with XG81, XG83 exhibited a higher RBD binding affinity and neutralization potency against wild-typed pseudovirus, variant pseudoviruses with mutated spike proteins, such as D614G, E484Q, and A475V, as well as the authentic SARS-CoV-2 virus. To explore potential broadly neutralizing antibodies, heavy and light chains from all 18 nAbs (16 published nAbs, XG81 and XG83) were cross-recombined, and some of the functional antibodies were screened and studied for RBD binding affinity, and neutralizing activity against pseudovirus and the authentic SARS-CoV-2 virus. The results demonstrated that several recombined antibodies had a more potent neutralization activity against variant pseudoviruses compared with the originally paired Abs. Taken together, the novel neutralizing antibodies identified in this study are a likely valuable addition to candidate antibody drugs for the development of clinical therapeutic agents against SARS-CoV-2 to minimize mutational escape.

## Introduction

The coronavirus disease 2019 (COVID-19) epidemic caused by the novel Severe Acute Respiratory Syndrome coronavirus type 2 (SARS-CoV-2) continues to spread on a global scale and has caused great damage to public health (1). SARS-CoV-2 spike protein (S) is a key factor in determining the viral invasion of susceptible cells through the receptor binding domain (RBD) that mediates the binding of the virus to host cell surface receptor, angiotensin-converting enzyme II (ACE2) (2). With the evolution of SARS-CoV-2, a portion of emergent S mutants exhibited an increased viral replication or infectivity in individuals, which has posed a strong impact on the pathogenesis and host immune system (3). SARS-CoV-2 D614G variant, which could produce higher infectious titers, has become dominant during the COVID-19 pandemic. Previous report suggested that D614G altered SARS-CoV-2 fitness and neutralization susceptibility, and that might be a challenge for therapeutic antibodies or vaccines (4). Recently, the mutation E484Q found in India variant (B.1.617) could escape the vaccine-induced humoral immune response (5). A475V variant at RBD has also been reported to become resistant to some nAbs (6). Currently, all of the COVID-19 vaccines in clinical trials were based on the original SARS-CoV-2 sequence (Wuhan-1); hence, to which degree virus mutations impact the effectiveness of COVID-19 vaccines remains unclear (7). Since multiple SARS-CoV-2 variants could escape from vaccine-induced humoral immunity, developing broadly protective interventions against the evolving virus has become an urgent need (8).

Neutralizing antibodies (nAbs) from humans are promising therapeutic agents against emerging viruses such as HIV, SARS, and Middle Eastern respiratory syndrome (MERS) and still remain a high priority in clinical studies (9–12). Since the outbreak of SARS-CoV-2, many institutions and researchers have committed to identify nAbs from convalescent COVID-19 patients and more than 100 neutralizing antibodies have been generated (13, 14). It was reported that some SARS-CoV-2 variants could not only increase the infectivity and antigenicity but also become resistant to some nAbs and anti-serum containing polyclonal antibodies (6, 15, 16). A combination or cocktail therapy with multiple nAbs might be effective in clearing various different virus mutants and preventing SARS-CoV-2 resistance (17, 18). Particularly, antibody therapy might also improve the passive immunity against viral infection in severe symptomatic patients or those whose immune system was weak or not able to elicit an effective immune response after infection or vaccination (19). However, the manufacturing costs to produce neutralizing antibodies and the inconsistent half-life of different antibodies and rapid emergency of SARS-CoV-2 variants might also limit the usage of an antibody cocktail therapy. Furthermore, escape mutants might be selected under pressure of antibody existence. Antibody therapy has showed a remarkable effect on the control of the epidemic; however, it is important to discover a more novel antibody against virus variants, especially those escaped viral mutants.

To screen for high-affinity humanized nAbs against SARS-CoV-2 in this study, RBD specific B cells screening, single cell cloning and sequencing were performed to obtain the antibody sequences. The affinity and neutralizing activity of the identified antibodies were verified following the antibody preparation and purification. Two nAbs, XG81 and XG83, were identified showing a high neutralization potency against different SARS-CoV-2 variants, including the ones with D614G, E484Q, or A475V mutations. Moreover, the recombined antibodies (rAbs) with heavy and light chains from different nAbs produced some new antibodies with a higher neutralization potency against E484Q or A475V mutations which showed immune escape from the original nAbs. These new antibodies identified in the present study show a potential value for the development of clinical therapeutic agents against mutated SARS-CoV-2.

## Methods and Materials

### Human Memory B Cell Enrichment and Plasmablast Activation

PBMCs from 25 convalescent COVID-19 patients were combined to enrich CD27^+^ memory B cells. Human memory B cell enrichment was carried out by the Human Memory B Cell Isolation Kit (Miltenyi Biotec, 130-093-546). Briefly, PBMCs were incubated with a cocktail of Biotin antibody (Miltenyi Biotec, 130-093-546, CD2, CD14, CD16, CD36, CD43, and CD235a) and then mixed with anti-Biotin conjugated beads to remove non-B cells. CD27, a member of the TNF-receptor family, is expressed on most memory B cells. The mixture was subsequently passed through the MACS LD column and followed by the positive isolation of memory B cells using CD27^+^ beads. Memory B cells were further cultured in an activation medium that contained CD40L-expressing feeder cells. The ratio of B cells to CD40L cells was 1:8,000 (20). Interleukin (IL)-2 (absin, abs04045) at 100 U/mL and IL-21 (absin, abs00826) at 50 μg/mL in 96-well plate for 3–5 days to be activated into plasmablast cells and culture supernatant was collected for anti-RBD IgG test. The study was approved by the Ethics Committee of the First Affiliated Hospital of USTC and all participants have provided a written informed consent.

### RBD Specific B Cells Isolation

SARS-CoV-2 spike protein specific antibodies in plasmablast cell culture supernatant were determined using enzyme-linked immunosorbent assay (ELISA). Briefly, 50 ng of recombinant SARS-CoV-2 RBD protein in 100 μl of PBS per well was coated on 96-well ELISA plates overnight at 4°C. The ELISA plates were blocked for 1 h with 100 μL of blocking buffer (5% non-fat dry milk in PBS). Ten-fold diluted culture supernatant was then added and incubated at 37°C for another one hour. After washing with PBST buffer (0.05% Tween 20 in PBS), the plates were incubated with anti-human IgG-HRP (Genscript) antibody for 30 min at 37°C. The plates were allowed to react for 10 min and stopped with 1 M HCl following PBST washing and adding of TMB buffer. The optical density was measured at OD_450_ nm. RBD-specific antibodies secreting cells with positive values were further loaded on a chip as single cell (Beacon, Genscript). The assay was processed by imported SARS-CoV-2 RBD specific beads (Berkeley Lights) and species specific anti-human IgG secondary antibody conjugated with Alexa flour (Invitrogen). After exposure to the Cy5 channel, the image was recorded and analyzed by the “Image Analyzer” software (Berkeley Lights) for positive hits.

### Single B Cell Sequencing

RNA from single B cell was purified using the AMPure RNA Clean XP kit and eluted directly into a 9 µL RT (reverse transcription) reaction. The RT reaction was performed by incubation at 42°C for 90 min, followed by 10 cycles of 50°C for 2 min, 42°C for 2 min, 75°C for 15 min according to the protocol of the manuscript (Berkeley Lights, 750-70002) and then left at 4°C until the next step. The 9 µL RT reaction product was added to a PCR mix using KAPA HiFi HotStart Readymix (kapa, KK2602) in total of 30 µL to amplify the complementary DNA (cDNA). The cDNA amplification was performed at 98°C for 3 min, followed by 20 cycles of 98°C for 15 s, 65°C for 30 s, 72°C for 6 min, with a final incubation at 72°C for 10 min, and stored at 4°C. A 1 µL portion of the cDNA amplification product was used to amplify the lambda and kappa chains in separate 15 µL reactions using primers in the antibody constant regions and forward primer to obtain the specific product for sequencing. The nucleotide sequences of the referenced primers were showed in **Table S3** (21). The product sequences were analyzed using the international ImMunoGeneTics information system^®^.

### Construction of Plasmids and Cell Lines

The SARS-CoV-2 specific antibody expression vectors were derived from the human immunodeficiency virus (HIV) neutralizing antibody VRC01 heavy and light chain expression plasmids (22) that contain the IgG1 constant regions of human from NIH with CMV promoter and Kana resistance. To express SARS-CoV-2-specific antibody, the sequences of heavy and light chain variable regions were separately cloned into the abovementioned antibodies expression vectors. SARS-CoV-2 surface glycoprotein gene (GenBank: MN_908947) with C-terminal 19 amino acids deletion was synthesized after codon-optimizing for *Homo sapiens*. Synthesized sequence added with restriction enzyme sites (*Xho* I and *Xba* I) was inserted into eukaryotic expression plasmid pcDNA3.1(+) to generate the envelope expression plasmids pcDNA3.1(+)-Opt-S. The lentiviral packaging plasmid pNL4-3 Luc+R-E- carrying an Env-defective, luciferase-expressed HIV-1 genome was gifted by Binlian Sun, Jianghan University. pTT5 vector and HEK293F cell line was generously gifted by Tengchuan Jin, University of Science and Technology of China. To construct the HEK293T-hACE2 cell line that stably expressed hACE2 for pseudovirus neutralization assay, the lentivirus containing hACE2 gene was transfected into 293T cells and were selected with 2 μg/mL of Puromycin.

### Expression and Purification of mAbs and SARS-CoV-2 RBD Protein

The amino acid sequence of 14 published SARS-CoV-2 nAbs, 1 SARS nAb, and 1 MERS nAb were downloaded from the PDB (protein data bank) database. Each antibody DNA sequence transformed from the amino acid sequence was codon-optimized for human cells. Heavy chain variable region sequences were synthesized with *Age* I and *Sal* I restriction enzyme sites, while light chain (κ or λ) variable region sequences were synthesized with *Age* I and *BsiW* I restriction enzyme sites. To express these nAbs in small scale, the synthesized sequences of paired heavy and light chain variable regions were separately inserted into expression vectors containing the IgG1 constant regions of human and co-transfected into 293T cells. Meanwhile, different originated heavy and light chain expression vectors were cross-paired and co-transfected into 293T cells. After 48 h post-transfection, cell supernatant containing expressed antibodies were collected to determine the RBD binding ability using ELISA assay.

**Figure 1:**
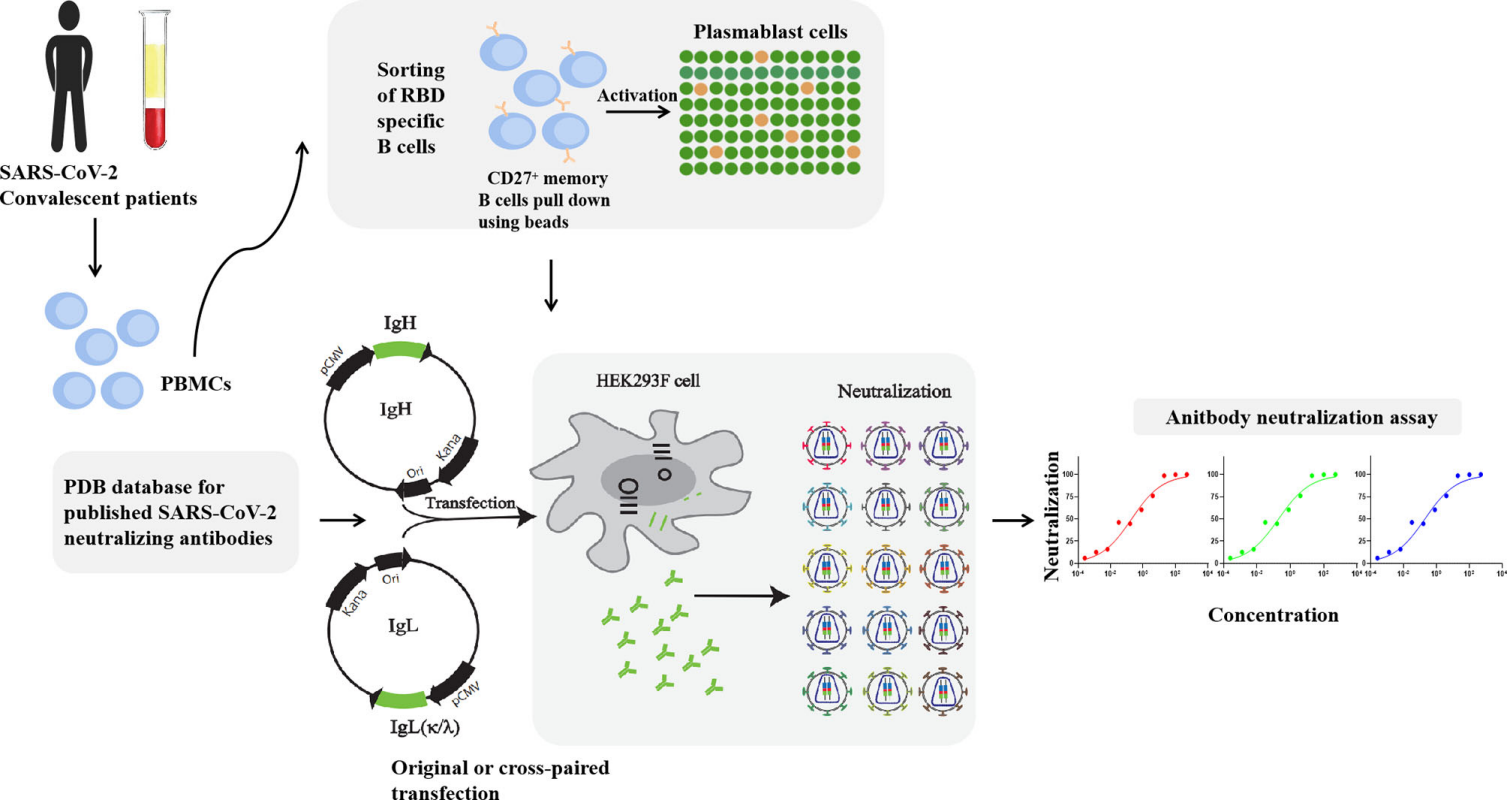
Illustration of antibodies production and neutralization assay. MAbs were produced by the co-transfection HEK293F cells with heavy and light chain expression vectors originated from a single sequence of RBD specific B cells. RAbs were produced by the co-transfection HEK293F cells with recombination of heavy and light chain from different single cells. Neutralization assay was performed by incubation of pseudovirus of live SARS-CoV-2 virus with serial diluted antibodies.

To explore the characters of antibodies, the corresponding antibodies with positive OD values (OD ratio > 2 relative to the negative control) detected by ELISA were expressed in a large scale. Equal amounts of heavy and light chain expression plasmids were co-transfected into HEK293F cells at a density of 2 × 10^6^ cells/mL at 37°C in a humidified 5% CO_2_ incubator rotating at 130 rpm. Briefly, to transfect 100 mL volume of HEK293F cells, 100 μg of heavy and 100 μg of light chain expression plasmids were prepared and mixed with 0.8 μg of polyethylenimine (PEI, Polyscience) and were added into cells. After 5 days, cell culture supernatants were collected and purified using Protein A column (GE Healthcare). Bound mAbs were washed with buffer containing 20 mM Na_2_HPO_4_, 150 mM NaCl (pH 7.0), eluted using 0.1 M acetic acid (pH 3.0-4.5) on ÄKTA pure (GE Healthcare), and resuspended into PBS by centrifugation with 10 kDa MWCO membrane centrifugal filter units (Millipore). The illustration of RBD specific B cells isolation, expression vectors construction, antibodies purification and identification are shown in **Figure 1**. The purity of antibodies was verified using SDS-PAGE (**Figure S1A**).

Sequence of RBD fused with human IgG1-Fc fragment were cloned into the pTT5 vector. A TEV (Tobacco Etch Virus) protease cleavage site was added between the RBD and IgG1-Fc sequence. Briefly, the Fc gene was amplified from antibody heavy chain expression plasmid using the forward primer: 5’-TCTAGAGGTTCTGAGAATCTTTATTTCCAAGGTTCTGGTTCT CCCAAATCTTGTGACAAAAC-3’ and the reverse primer: 5’-GCGGCCGCTTATTTACCCGGA GACAGGGAGA-3’ (underlined sequences are *Xba* I and *Not* I restriction enzyme sites, respectively). The PCR product encoding Fc with N’-TEV protease cleavage site was cloned into the pTT5 plasmid to generate pTT5-TEV-Fc vector. The gene encoding RBD (321–591) was amplified from spike gene of SARS-CoV-2 (Gene ID: MN908947) using the forward primer: 5’-GTCGACCAACCAACAGAATCTATTGT-3’ and the reverse primer: 5’- TCTAGA AGAACATGGTGTAATGTCAA-3’ (underlined sequences are *Sal* I and *Xba* I restriction enzyme sites, respectively). The PCR product was cloned into the pTT5-TEV-Fc plasmid to generate the pTT5-RBD-TEV-Fc vector. The resultant vector was transfected into HEK293F cells using PEI and fusion protein RBD-Fc was purified from cell supernatants 3 days post-transfection using Protein A column as described above. To remove the Fc tag, 20 mM TEV, 1 mM DTT, and 1 M Tris were added into the purified fusion protein. The mixture was incubated at 4°C overnight and purified using Protein A column. Fc-free recombinant proteins were collected and the purity of proteins was verified by SDS-PAGE (**Figure S1B**).

### ELISA Quantification for RBD Binding Ability

SARS-CoV-2 RBD protein at 2 ng/μL was coated onto ELISA plates overnight at 4°C. After washing and blocking, 100 μL of cell culture supernatant containing mAbs from 293T cells were added to each well and incubated at 37°C for 2 h. Plates were washed and incubated with anti-human IgG (H+L)/HRP (Abcam) for 1 h at 37°C. TMB (Beyotime) substrate was added and reacted under dark and optical density (OD) was measured at 450 nm.

### Antibodies Binding Affinity Measurement

The dissociation coefficient was detected using surface plasmon resonance (SPR). Briefly, RBD was coated on a CM5 SA sensor chip (GE Healthcare) by covalent bonding at a level of 60 response units (RU) using a Biacore T200 (GE Healthcare). Running buffer was composed of PBS with the regeneration solution composed of 50 mM NaOH. Purified mAbs were diluted in serial concentrations and injected at a flow rate of 30 μL/min. All injections were performed with an association time of 300 s and a dissociation time of 600 s. Data were fit to a 1:1 binding model using the Biacore Evaluation Software. All curves were plotted using GraphPad Prism 8.3.

### Competitive ELISA Assay

The antibody competition assay was performed to identify the targeted epitope of the two mAbs. The two mAbs were biotinylated using Sulfo-NHS-LC-LC-Biotin (Thermo) overnight at 4°C separately. RBD at 2 ng/μL (200 ng of RBD protein in 100 uL of PBS per well) was coated on ELISA plates overnight at 4°C. The plates were washed with PBST followed by blocked with 5% defatted milk for 2 h at 37°C. Meanwhile, nine previously published SARS-CoV-2 nAbs with known targeted epitopes at 4-fold serially diluted in PBS with the initial dilution of 20 μg/mL (concentration range of 20 μg/mL to 0.078 μg/mL) were added into wells and followed by an equal volume of antibody-Biotin diluted at 1 μg/mL. After 1 h of incubation and three times washing, the plates were incubated with HRP conjugated Streptavidin (1:5,000, Beyotime) for 30 min at 37°C. Next, the plates were washed with PBST and treated with the TMB buffer. After 10 min, 50 μL of stop buffer was added to stop the reaction, and the absorbance was read at OD_450_ nm. The inhibition rate was calculated by comparing to the negative control well with only antibody-Biotin added.

### Pseudotyped Virus Neutralization Assay

Pseudoviruses with spike mutation were produced by co-transfection HEK293T cells with pNL4-3 Luc+R-E- and plasmids encoding various spike mutation, while Wuhan reference pseudovirus was produced with pNL4-3 Luc+R-E- and pcDNA3.1(+)-Opt-S. The supernatant was harvested at 48 h post-transfection, passed through a 0.45-μm filter, and centrifuged at 800 × g for 10 min to remove cell debris. Cell supernatant was collected and stored at -80°C. The 293T cells stably expressing hACE2 were seeded in 96-well plates at a density of 1.0 × 10^4^/well 24 hours prior to the assay. The various diluted mAbs (5-fold serial dilution at an initial concentration of 100 μg/mL, 50 μL aliquots) were mixed with an equal volume of SARS-CoV-2 pseudovirus with luciferase units of 1 × 10^5^ and incubated at 37°C for 1 h, which were added to the HEK293T-hACE2 cell wells. Negative control wells were supplied with 100 μL of DMEM while positive control wells were supplied with 50 μL of DMEM and 50 μL of pseudovirus. After 48 hours of incubation at 37°C with 5% CO_2_, culture supernatants were removed and the values of RLU were measured by the Britelite plus Reporter Gene Assay System (PerkinElmer). The inhibition rate was calculated by comparing the OD value to the positive control wells. Fifty percent inhibitory concentration (IC_50_) was determined by a four-parameter logistic regression using GraphPad Prism 8.0.

### Authentic Virus Neutralization Assay

The neutralization assay of authentic SARS-CoV-2 was detected using CPE (cytopathic effect) assay. The CPE was recorded when virus caused morphological changes in the Vero E6 cells. Vero E6 cells were seeded in monolayers in 96-well plates and incubated at 37°C overnight. Four-fold dilution of mAbs with an initial concentration of 300 μg/mL were mixed with the same volume of SARS-CoV-2 authentic virus at 100 TCID50 and incubated for 1 h at 37°C. The mAbs and virus mixtures were then transferred to Vero E6 cell wells and incubated at 37°C for 1 h. One hour later, the supernatant was removed and 200 μL of DMEM with 2% FBS was added to cells. After the incubation of the cells with the mixture for 5 days at 37°C, CPE caused by the infection was recorded. The authentic SARS-CoV-2 virus was isolated from a COVID-19 patient from Anhui Province. All experiments associated with the authentic virus were conducted in Biosafety Level 3 (BSL-3) laboratory in Anhui Provincial Center for Disease Control and Prevention. All experiments were complied with the biosecurity and institutional safety.

### Statistical Analysis

IC_50_ that is defined as the dilution at which the RLU values were reduced by 50% compared with the pseudovirus control wells was calculated in GraphPad Prism 8 (GraphPad Software). The difference of the Log_10_ IC_50_ of two independent groups was tested for statistical significance with a Mann-Whitney U test in GraphPad Prism 8. The KD values were calculated using 1:1 binding model in Origin 8.0 (OriginLab).

## Results

### Single B Cell Antibody Cloning and Sequence Analysis

**Figure 2:**
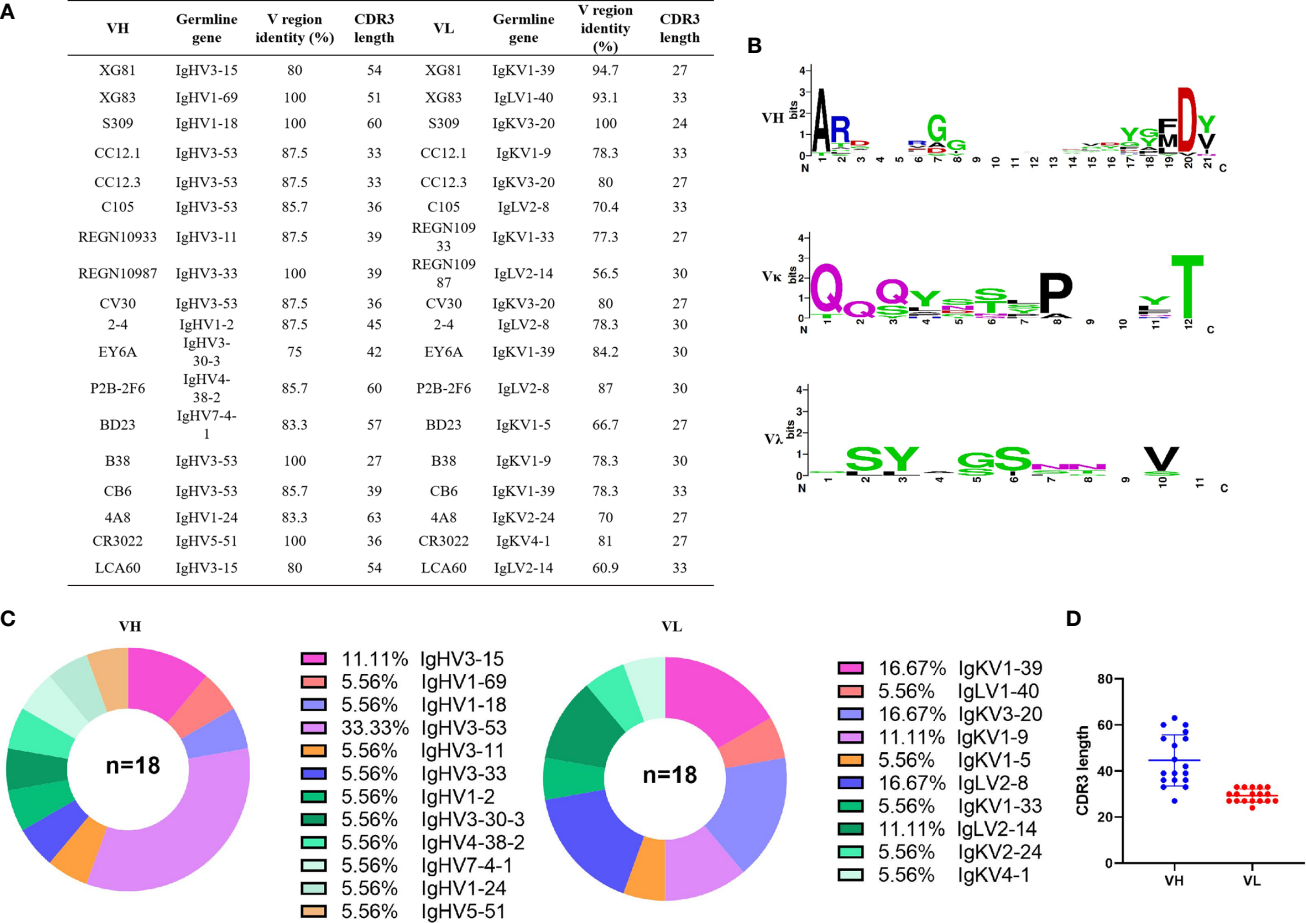
Sequencing analysis of germline and CDR3 region of antibodies. **(A)** Germline, CDR3 length of nucleotides sequence, and variable region identity of the heavy and light chains of the 18 mAbs. **(B)** Amino acids sequence alignment of CDR3 region using THE MEGA 6.0 and Weblogo web server (http://weblogo.berkeley.edu/logo.cgi). **(C)** Distribution of variable germline in heavy and light chains of the 18 mAbs. **(D)** CDR3 amino acid length of VH and VL of the 18 mAbs. CDR3, complementary determining region 3.

To isolate the monoclonal antibodies, we combined the PBMCs from 25 convalescent COVID-19 patients to enrich CD27^+^ memory B cells and activate memory B cells into plasmablast cells. We isolate RBD specific B cells by detecting the SARS-CoV-2 spike protein specific antibodies in the plasmablast cell culture supernatant using ELISA. Cells with positive OD values were used for further single cell sequencing. We finally obtained two sequences of mAbs and they were detected positive for RBD binding after cloning and expression (sequences please see extended data, **Table S1**). Furthermore, we searched the PBD (protein bank data) database and downloaded 14 IgG heavy (IgH) and light (IgL, κ or λ) sequences of SARS-CoV-2 nAbs (S309, CC12.1, CC12.3, C105, REGN10933, REGN10987, CV30, 2-4, EY6A, P2B-2F6, BD23, B38, CB6, and 4A8), one SARS-CoV nAb (CR3022), and one MERS nAb (LCA60). The CDR3 region, length of nucleotides sequence, variable region identity, and germline feature of IgH and IgL of the total 18 antibodies were analyzed using Igblast (**Figures 2A, C**). **Figure 2C** is the pie chart that represents the germline distribution of heavy and light chains of the 18 mAbs. As shown in **Figure 2C**, about 33.33% of the heavy chains belong to the IgHV3-53 group, and 11.11% belong to the IgH3-15 group, while others are distributed to 10 variable germlines evenly. As for the light chains, 16.67% belonged to the IgKV1-39, IgKV3-20, and IgLV2-8 germlines, and 11.11% belonged to the IgKV1-9 and IgLV2-14 germlines. The average CDR3 length for IgH was 44.67 and the average IgL CDR3 length was 29.33 (**Figure 2D**). The length of CDR3 region of XG81 was 54 and the CDR3 region length of XG83 was 51, which were both above the average CDR3 length. The length of CDR3 region of XG81 IgL was 27, while the CDR3 length of XG83 IgL was 33 and the value was also above the average length. The heavy chain variable region of XG81 and XG83 belongs to the V_H_ 3-15 and V_H_1-69 families while the corresponding light chains belong to the V_k_1-40 and V_L_1-39 families. The amino acid sequences variety and alignment for CDR3 region of IgH, Igκ, and Igλ are presented in **Figure 2B**. The size of the letter represents the frequency of the occurrence of a certain amino acid in the 18 mAbs at the same site, suggesting the differences in the amino acid sequence among these antibodies (**Figure 2B**). The alignment of the 18 mAbs for CDR1, CDR2, and CDR3 regions is shown in the extended data (**Figure S2**).

### Binding Profiles of Originally Paired Monoclonal Antibodies and rAbs to SARS-CoV-2 RBD Protein

**Figure 3:**
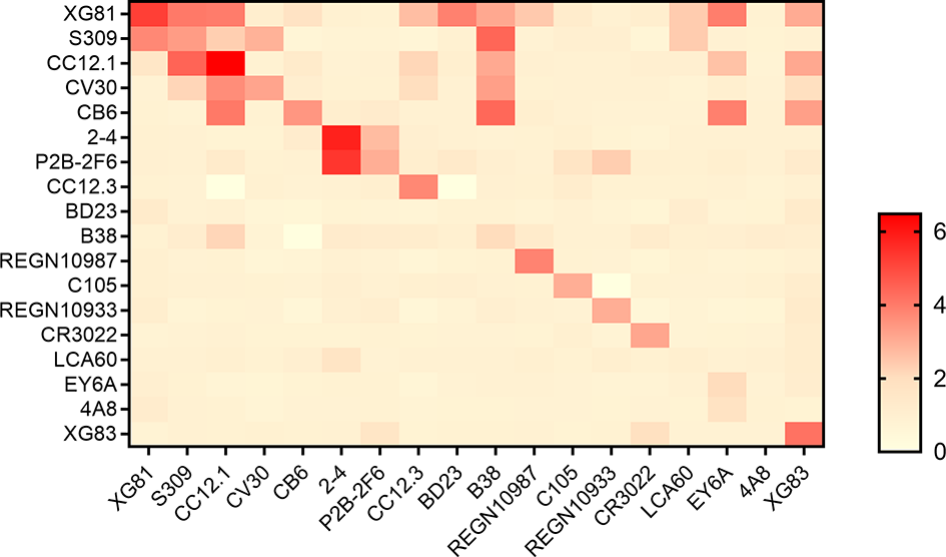
Binding profiles of antibodies to RBD. Heatmap showed the binding of antibodies to RBD using ELISA. Color in the boxes indicated the values of detecting antibodies binding to RBD relative to the negative control and the ratio of an OD value > 2 was considered positive. Experiments were repeated twice and data were shown as a representative experiment.

Heavy and light chains from all 18 nAbs were recombined to form recombined antibodies (rAbs) and subjected to studies for their RBD binding characteristics. All 324 transfection supernatants from 293T cells were assayed using ELISA for the RBD binding affinity. The ratio of the OD value to the negative control over than 2 was considered positive binding to RBD (extended data, **Table S2**). As shown in **Figure 3**, in total, 44 out of 324 antibodies were tested positive of RBD binding. Of the 18 originally paired mAbs, 15 were measured positive for RBD and the remaining 3 mAbs were negative, including one against MERS (LCA60), one against N-terminal of SARS-CoV-2 spike protein (4A8), and another one binds the “down” RBD in protomer B (BD23). Interestingly, of the 306 rAbs, 29 antibodies also showed positive for RBD binding test, suggesting that the cross-paired expression form different origins might also be one potential tool to produce binding antibody.

### Binding Activities of Antibodies to RBD

**Figure 4:**
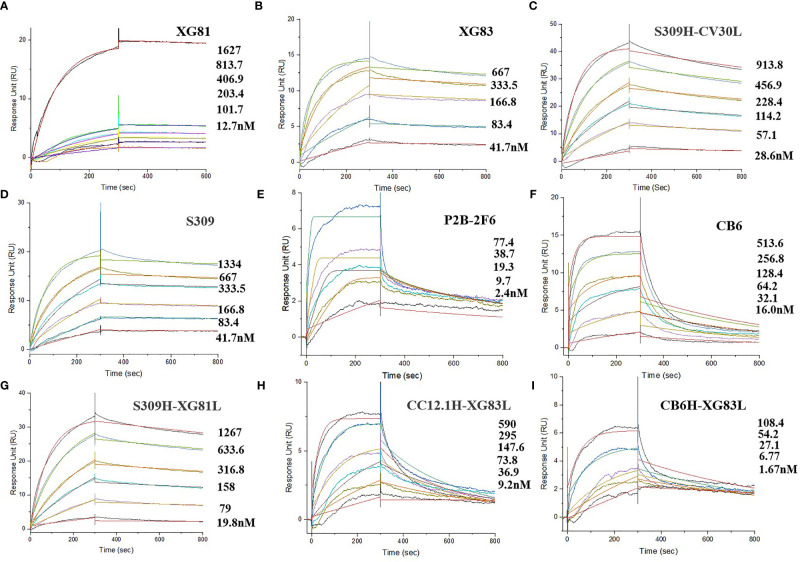
Affinity abilities of antibodies to SARS-CoV-2 RBD. Surface plasmon resonance (SPR) demonstrated the binding and dissociation kinetics of the 9 antibodies (**A**: XG81; **B**: XG83; **C**: S309H-CV30L; **D**: S309; **E**: P2B-2F6; **F**: CB6; **B**: S309H-XG81L; **H**: CC12.1-XG83L; **I**: CB6H-XG83L) against S.

Through the initial screening of live SARS-CoV-2 virus neutralization test, we selected nine antibodies with a relatively better neutralizing ability, including XG81, XG83, 3 SARS-CoV-2 nAbs (S309, P2B-2F6, and CB6), and four rAbs (S309H-CV30L, S309H-XG81L, CC12.1H-XG83L, and CB6H-XG83L) to determine the binding affinity using surface plasmon resonance (SPR). The dissociation constant (KD) for the nine antibodies ranged from 10^-8^ to 10^-10^ M (**Figures 4A–I**). XG81 showed the KD of 10.5 nM while XG83 showed the KD of 5.68 nM. S309, P2B-2F6, and CB6 exhibited KD at 6.04, 0.81, and 43.8 nM, respectively. The rAbs also exhibited RBD binding affinity with the KD ranging from 0.58 to 21.1 nM. Interestingly, one of the rAbs, CB6H-XG83L, showed the highest binding affinity to RBD among the 9 antibodies with the KD at 0.58 nM (**Table 1**).

**Table 1:** Affinity and neutralization characteristics of 10 antibodies.

Antibody	KD (RBD, nM)	IC_50_ (μg/mL)				
		Wuhan	D614G	E484Q	A475V	Authentic Virus
XG81	10.5	0.556	2.431	0.010	1.531	75.000
XG83	5.68	0.069	0.174	0.033	0.057	3.443
S309H-CV30L	19.5	0.501	0.318	0.129	0.038	4.404
S309	6.04	0.198	0.006	0.029	0.014	0.628
P2B-2F6	0.81	0.009	0.020	0.049	0.044	37.500
CB6	43.8	0.005	0.005	0.017	0.155	0.628
S309H-XG81L	21.1	0.015	0.022	0.144	0.027	1.248
CC12.1H-XG83L	12.9	0.005	0.006	0.009	0.052	1.172
CB6H-XG83L	0.58	0.036	0.004	0.004	0.010	1.101
CV30	/	0.020	0.005	0.006	0.075	0.293

### Neutralization Properties of RBD Specific Antibodies

**Figure 5:**
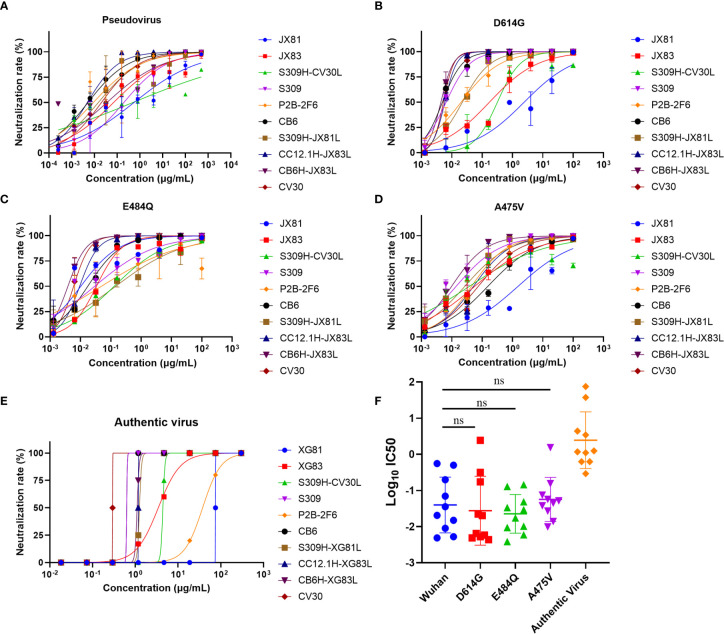
Neutralizing capacities of antibodies for pseudovirus and authentic virus. **(A)** Neutralization of antibodies to SARS-CoV-2 pseudovirus in ACE2-293T cells. **(B)** Neutralization to D614G mutant pseudovirus. **(C)** Neutralization to E484Q mutant pseudovirus. **(D)** Neutralization to A475V mutant pseudovirus. **(E)** Neutralization of antibodies to authentic SARS-CoV-2 in Vero-E6 cells. **(F)** Log_10_ IC_50_ values of antibodies to four pseudoviruses and live virus. The difference of Log_10_ IC_50_ values in two groups was tested for statistical significance with a Mann-Whitney U test in GraphPad Prism 8. ^ns^ *p* > 0.05. Experiments were repeated twice and data were shown as Mean ± SD of a representative experiment. ns, no significant difference.

To better evaluate the neutralization properties of some original mAbs and rAbs, we further selected 10 antibodies to measure the inhibition rate of pseudovirus and live SARS-CoV-2 virus. For the original Wuhan reference pseudovirus, the XG83 showed IC_50_ of 0.069 μg/mL, and XG81 showed the IC_50_ of 0.556 μg/mL. The S309, P2B-2F6, and CB6 showed the IC_50_ at 0.198, 0.009, and 0.005 μg/mL and the CV30 showed the IC_50_ at 0.020 μg/mL, respectively (**Figure 5A** and **Table 1**). As we mentioned above, the dominated variant D614G could increase infectivity while E484Q and A475V exhibited resistance to some nAbs. Our study showed that all the original mAbs and rAbs could effectively neutralize the three pseudovirus variants with a mutated S protein containing D614G, E484Q, or A475V mutation (**Figures 5B–D** and **Table 1**). We also compared the neutralization activities between the mutant and Wuhan reference pseudovirus. The IC_50_ ratio higher than 1 indicated the decreased ability of antibodies in neutralizing mutant pseudovirus relative to Wuhan reference pseudovirus. The antibodies that targeted E484Q or A475V residue of RBD all showed increased IC50 values on mutant pseudovirus (ratio > 1, **Table 2**). Interestingly, some rAbs antibodies such as CB6H-XG83L and S309H-CV30L were granted a significantly higher neutralizing ability to mutant pseudoviruses when compared with the original mAbs (ratio < 1, **Table 2**).

**Table 2:** Comparison of the neutralization ability of 10 antibodies for mutant and Wuhan pseudotyped virus.

Antibody	E484Q residue		IC_50_ ratio (E484Q/Wuhan)	A475V residue		IC_50_ ratio (A475V/Wuhan)
	VH	VL		VH	VL	
XG81	/	/	0.018	/	/	2.754
XG83	/	/	0.478	/	/	0.816
S309H-CV30L	–	–	0.257	–	–	0.076
S309	–	–	0.148	–	–	0.070
P2B-2F6	–	+	5.410	–	–	4.920
CB6	–	–	3.306	+	–	29.440
S309H-XG81L	–	/	9.639	–	/	1.800
CC12.1H-XG83L	–	/	1.918	–	/	10.596
CB6H-XG83L	–	/	0.108	+	/	0.276
CV30	–	–	0.290	+	–	3.658

The IC_50_ ratio higher than 1 indicated the decreased ability of antibodies in neutralizing the mutant pseudovirus relative to Wuhan reference pseudovirus.

To confirm the neutralization activities of the antibodies, we further performed the cytopathic effect (CPE) inhibition assay with the authentic SARS-CoV-2 virus. The XG81 and XG83 showed IC_50_ of 75.00 and 3.443 μg/mL, respectively, and CV30 showed the lowest IC_50_ of 0.293 μg/mL, followed by S309 and CB6 with IC_50_ of 0.628 μg/mL (**Figure 5E**). The rAbs all showed their neutralization abilities against both pseudovirus and live SARS-CoV-2 virus, and the neutralization potencies were mostly between or close to the original antibodies. As shown in **Figure 5F**, the Log_10_ IC50 values of the 10 antibodies against Wuhan pseudotyped virus were higher than that of D614G and E484Q pseudoviruses, but lower than A475V, suggesting that G614G and E484Q were more susceptible to neutralization while A475V showed a somewhat escape from neutralization. Additionally, the authentic virus showed higher Log_10_ IC50 value, exhibiting more difficult to be neutralized than the pseudovirus.

### Competitive Characteristics for Antibodies

**Figure 6:**
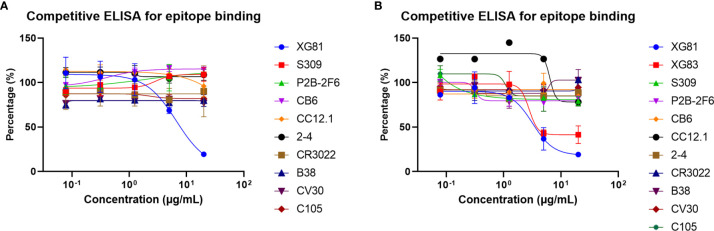
Competition characteristics of XG81 and XG83. **(A)** The competitive capacity of XG81 with nine SARS-CoV-2 nAbs that was indicated by the level of response unit relative to the negative control with the increase of nAbs concentration. XG81 was used as a positive control. **(B)** The competitive capacity of XG83 with XG81 and another nine SARS-CoV-2 nAbs. XG83 itself was used as a positive control.

A competition ELISA was further performed for the two nAbs to determine if there were overlapping epitopes between different nAbs. Nine previously reported SARS-CoV-2 RBD nAbs (S309, P2B-2F6, CB6, CC12.1, 2-4, CR3022, B38, CV30, and C105) were used as references while the two nAbs (XG81 and XG83) themselves were used as positive controls. With the decrease change of the concentration of the nine nAbs, the absorbance percentage showed no obvious change, suggesting that they target new epitopes on the spike protein (**Figures 6A, B**). With the decreasing change of the concentration of XG83, the absorbance percentage of XG81 increased obviously, which suggests that they have a competitive effect with each other and shared similar epitopes of SARS-CoV-2 (**Figure 6B**).

## Discussion

The rapid spread of SARS-CoV-2 has caused great threat globally. Despite the fact that the vaccines and antiviral drugs has been widely used on the market, the protective effectiveness remains uncertain against the rapidly emerging variants. Therefore, isolation of nAbs still remains an important strategy for the clinical treatment of SARS-CoV-2 infection. We have identified two SARS-CoV-2 nAbs from plasmablast B cells of convalescent COVID-19 patients that both recognize the RBD region and neutralize live virus. One of the nAbs, XG83, showed a longer CDR3 length of IgH and IgL, and belonged to an independent germline. Since some variants, including A475V, E484Q, and D614G, have been reported to show a decreased sensitivity to neutralizing nAbs or an increased infectivity (6, 15), we next investigated the neutralizing activity of these antibodies against the mutant pseudoviruses. Both XG81 and XG83 could effectively neutralize against Wuhan, E484Q, A475V, and D614G pseudoviruses. Meanwhile, XG83 had a higher affinity to SARS-CoV-2 RBD, and a higher potency to neutralize Wuhan, mutant pseudoviruses, and authentic SARS-CoV-2 virus. Competitive ELISA revealed that XG81 and XG83 shared similar targets but different from other nAbs, demonstrating new binding sites for either of the two antibodies. The result suggests that XG83 could be a potential broadly protective monoclonal antibody with new antigenic targets.

The rAbs also showed binding affinity to RBD and neutralizing ability against both pseudovirus and authentic virus. One of the rAbs, CB6H-XG83L, showed the least KD value, which means the highest binding affinity to RBD. Since A475V is located within the binding sites of CB6, the IC50 value of A475V pseudovirus is 29.4 times relative to Wuhan pseudovirus. Similarly, the IC50 of CV30 in neutralizing A475V is 3.7 times relative to Wuhan pseudovirus, while the IC50 of P2B-2F6 is 5.4 times targeting E484Q compared to Wuhan pseudovirus. Interestingly, when compared with the originally paired mAbs, the rAb, CB6H-XG83L, effectively enhanced its neutralizing activity against A475V pseudoviruses so as to avoid the immune escape of the mutation (**Table 2**). The other rAb, S309H-CV30L, also showed a decreased IC50 ratio with a higher potency of neutralization compared with the original CV30 antibody (**Table 2**). Pseudoviruses test with single amino acid mutation could be helpful for evaluating the factors and exact mutation sites that influenced antibodies neutralizing activity, and highlights the key to modify and produce antibodies with higher neutralizing potency and breadth.

SARS-CoV-2 variants with various S mutations possibly arise over the global infected individuals along with virus evolution, which leaves a challenge and pressure for antiviral therapy. Studies proposed that the non-competing antibody cocktail therapy present a promising approach against SARS-CoV-2 variants with spike protein mutations (23). However, the identification of neutralizing mAbs with simultaneous effectiveness requires further study. Our study provided new insights to antibody therapy, that is recombination of heavy and light chains from various nAbs targeting specific antigenic sites, which could contribute to the further designing of antibody-based COVID-19 therapeutics. The underlying mechanisms of the rAbs in enhancing the neutralizing activity might be the overlapping of critical residues in RBD mutants. Notably, identification of an ideal combination could be essential for the design of rAbs expression. Since the rAbs that showed neutralizing ability was a new and interesting discovery of our study, the danger of these rAbs still remains unclear now. The potential risks might include off target or cross immune response. Therefore, the stability, exact targeting sites, and underlying mechanisms rAbs in enhancing neutralization potency remain an interesting topic and warrant future study. Besides, more SARS-CoV-2 variants with single or multiple mutations and currently circulating strains should be concerned in investigating broadly protective effectiveness of XG83 mAb and rAbs.

In conclusion, our data reports a fully human neutralizing mAb, XG83, could be used as a potential therapeutic agent for clinical treatment of SARS-CoV-2 infection. Combined expression of rAbs from different origination may function as additional potent neutralizing antibodies and be used to fight against various escaping mutants.

## Data Availability Statement

The datasets presented in this study can be found in online repositories. The names of the repository/repositories and accession number(s) can be found below: NCBI Genbank, accession numbers: MZ668598, MZ668599, MZ668600, MZ668601.

## Ethics Statement

The study was approved by the Ethics Committee of the First Affiliated Hospital of USTC and all participants provided their written informed consent to participate in this study.

## Author Contributions

TJ, SZ, and YG designed the study. JX, CD, JH, YZ, SN, XZ, QC, JW, LH, HH, WL, and HM performed the experiments. JX analyzed the data and wrote the manuscript. All authors contributed to the article and approved the submitted version.

## Funding

This work is supported by Postdoctoral Research Foundation of China (2021M693076; 2020M670084ZX), Key Research and Development Project of Anhui Province (202104j07020042; 202104a07020032), Special Project for Emergency Scientific and Technological Research on New Coronavirus Infection (YD9110002001), Emergency Research Project of Novel Coronavirus Infection of Anhui Province (202004a07020002; 202004a07020004), the Fundamental Research Funds for the Central Universities (WK9110000166; WK9110000167), and the Hefei Comprehensive National Science Center.

## Conflict of Interest

The authors declare that the research was conducted in the absence of any commercial or financial relationships that could be construed as a potential conflict of interest.

## Publisher’s Note

All claims expressed in this article are solely those of the authors and do not necessarily represent those of their affiliated organizations, or those of the publisher, the editors and the reviewers. Any product that may be evaluated in this article, or claim that may be made by its manufacturer, is not guaranteed or endorsed by the publisher.
