# Supplementary material for: Novel Monoclonal Antibodies and Recombined Antibodies Against Variant SARS-CoV-2
Source: Front Immunol. 2021 Aug 30;12:715464. doi: 10.3389/fimmu.2021.715464 (PMC8442604; doi:10.3389/fimmu.2021.715464)
Supplement: Supplementary file 1 [file DataSheet_1.docx]

**Figure S1. SDS-PAGE analysis for the purified proteins used in this study. A. Purified antibodies from HEK293F cell culture supernatants using Protein A columns. B. Purified RBD-Fc fusion from Protein A column and the RBD protein that digested using TEV protease.**


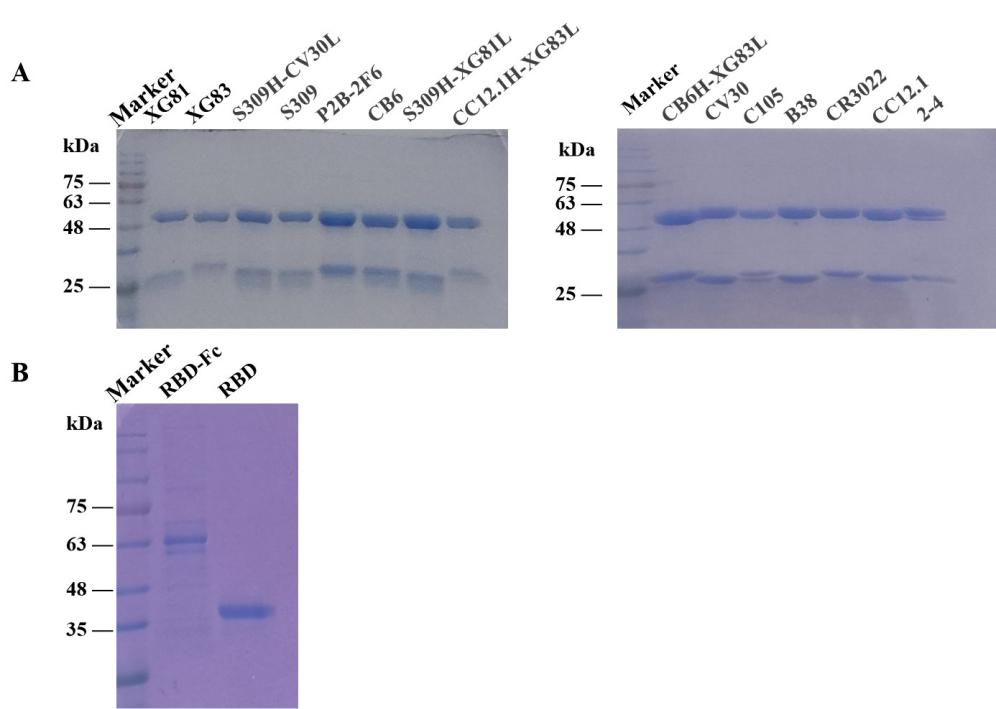


**Figure S2. Sequence alignment of the 18 antibodies. We obtained the sequences of the 16 published neutralizing antibodies from PBD website. A. Alignment of the heavy chain variable domain sequences of the 18 antibodies. B. Alignment of the kappa light (κ) chain variable domain sequences of the 12 antibodies. C. Alignment of the lambda light (λ) chain variable domain sequences of the 6 antibodies.**

**
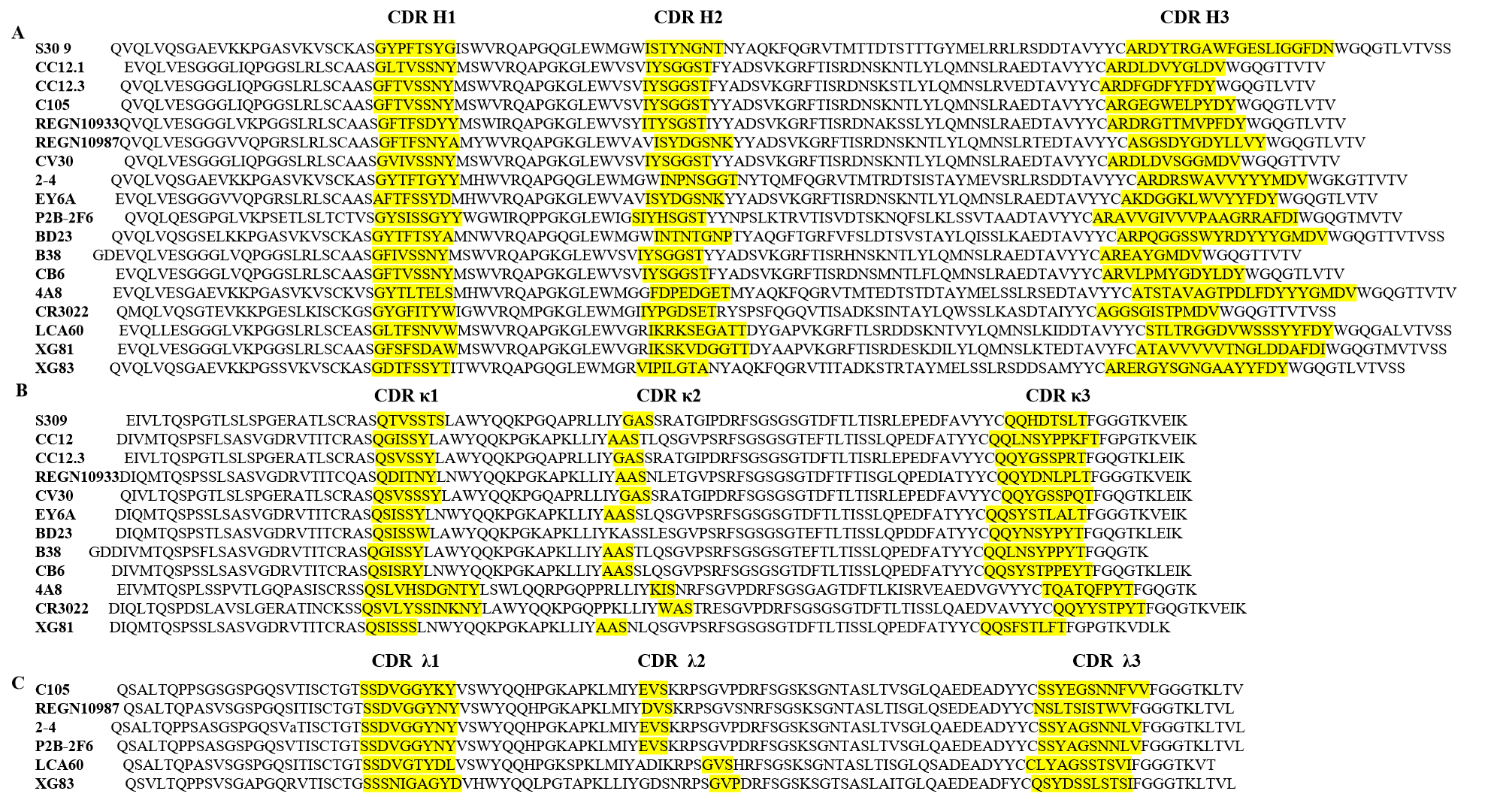
**

**Table S1. Nucleotide and amino acid sequences of VH and VL of XG81 and XG83 variable regions.**

| **ID** | **Nucleotide sequence** | **Amino acid sequence** |
| --- | --- | --- |
| **XG 81-VH** | ATGGAGTTTGGGCTGAGTTGGATTTTCCTTGCTGCTATTTTAAAAGGTGTCCAGTGTGAGGTGCAGCTGGTGGAGTCTGGGGGAGGCTTGGTAAAGCCTGGGGGGTCCCTGAGACTCTCCTGTGCAGCCTCTGGATTCAGTTTCAGTGACGCCTGGATGAGCTGGGTCCGCCAGGCTCCAGGGAAGGGGCTGGAATGGGTTGGCCGTATTAAAAGTAAAGTTGATGGTGGGACAACAGACTACGCTGCACCCGTGAAAGGCAGATTCACCATCTCAAGAGATGAATCAAAAGACATATTGTATCTGCAAATGAACAGCCTCAAAACCGAGGACACAGCCGTCTATTTTTGTGCCACTGCTGTAGTGGTGGTAGTTACTAATGGTCTTGATGATGCTTTTGATATCTGGGGCCAGGGGACAATGGTCACCGTCTCTTCA | MEFGLSWIFLAAILKGVQCEVQLVESGGGLVKPGGSLRLSCAASGFSFSDAWMSWVR  QAPGKGLEWVGRIKSKVDGGTTDYAAPVKGRFTISRDESKDILYLQMNSLKTEDTAV  YFCATAVVVVVTNGLDDAFDIWGQGTMVTVSS |
| **XG 81-VL** | ATGAGGGTCCCCGCTCAGCTCCTGGGGCTCCTGCTACTCTGGCTCCGAGGTGCCAGATGTGACATCCAGATGACCCAGTCTCCATCTTCCCTGTCTGCATCTGTAGGAGACAGAGTCACCATCACTTGCCGGGCAAGTCAGAGCATTTCCAGCTCTTTAAATTGGTATCAACAAAAACCAGGGAAAGCCCCTAAGCTCCTGATCTATGCTGCGTCCAATTTGCAAAGTGGGGTCCCATCAAGGTTCAGTGGCAGTGGATCTGGGACAGATTTCACTCTCACCATCAGCAGTCTGCAACCTGAAGATTTTGCAACTTACTACTGTCAACAGAGTTTCAGTACCCTTTTCACTTTCGGCCCTGGGACCAAAGTGGATCTCAAA | MRVPAQLLGLLLLWLRGARCDIQMTQSPSSLSASVGDRVTITCRASQSISSSLNWYQQK  PGKAPKLLIYAASNLQSGVPSRFSGSGSGTDFTLTISSLQPEDFATYYCQQSFSTLFTFGPG  TKVDLK |
| **XG 83--VH** | ATGGACTGGACCTGGAGGTTCCTCTTTGTGGTGGCAGCAGCTACAGGTGTCCAGTCCCAGGTCCAGCTGGTGCAGTCTGGGGCTGAGGTGAAGAAGCCTGGGTCCTCGGTGAAGGTCTCCTGCAAGGCTTCTGG  AGACACCTTCAGCAGCTATACTATCACCTGGGTGCGACAGGCCCCTGGACAAGGGCTTGAGTGGATGGGAAGGGTCATCCCTATCCTTGGTACAGCAAACTACGCACAGAAGTTCCAGGGCAGAGTCACGATTACCGCGGACAAATCCACGAGAACAGCCTACATGGAGTTGAGCAGCCTGAGATCTGACGACTCGGCCATGTATTACTGTGCGAGAGAACGTGGATATAGTGGCAACGGGGCCGCCTACTACTTTGACTACTGGGGCCAGGGAACCCTGGTCACCGTCTCCTCG | MDWTWRFLFVVAAATGVQSQVQLVQSGAEVKKPGSSVKVSCKASGDTFSSYTITWVRQ  APGQGLEWMGRVIPILGTANYAQKFQGRVTITADKSTRTAYMELSSLRSDDSAMYYCAR  ERGYSGNGAAYYFDYWGQGTLVTVSS |
| **XG 83--VL** | ATGGCCTGGTCTCCTCTCCTCCTCACTCTCCTCGCTCACTGCACAGGGTCCTGGGCCCAGTCTGTGCTGACGCAGCCGCCCTCAGTGTCTGGGGCCCCAGGGCAGAGGGTCACCATCTCCTGCACTGGGAGCAGCTCCAACATCGGGGCAGGTTATGATGTACACTGGTACCAGCAGCTTCCAGGAACAGCCCCCAAACTCCTCATCTATGGTGACAGCAATCGGCCCTCAGGGGTCCCTGACCGATTCTCTGGCTCCAAGTCTGGCACCTCAGCCTCCCTGGCCATCACTGGGCTCCAGGCTGAGGATGAGGCTGATTTTTACTGCCAGTCCTATGACAGCAGCCTGAGTACTTCGATATTCGGCGGAGGGACCAAACTGACCGTCCTA | MAWSPLLLTLLAHCTGSWAQSVLTQPPSVSGAPGQRVTISCTGSSSNIGAGYDVHWYQQ  LPGTAPKLLIYGDSNRPSGVPDRFSGSKSGTSASLAITGLQAEDEADFYCQSYDSSLSTSIFGG  GTKLTVL |

**Table S2. Ratio of OD values of antibodies relative to negative control.**

|  | **XG81** | **S309** | **CC12.1** | **CV30** | **CB6** | **2-4** | **P2B-2F6** | **CC12.3** | **BD23** | **B38** | **REGN10987** | **C105** | **REGN10933** | **CR3022** | **LCA60** | **EY6A** | **4A8** | **XG83** |
| --- | --- | --- | --- | --- | --- | --- | --- | --- | --- | --- | --- | --- | --- | --- | --- | --- | --- | --- |
| **XG81** | 5.21 | 4.04 | 3.99 | 1.07 | 1.74 | 0.89 | 0.83 | 2.67 | 3.89 | 3.11 | 2.44 | 1.21 | 0.87 | 1.07 | 2.39 | 3.96 | 0.81 | 3.04 |
| **S309** | 3.74 | 3.36 | 2.33 | 2.91 | 0.61 | 0.73 | 0.71 | 0.60 | 0.89 | 4.44 | 0.80 | 0.97 | 0.97 | 0.61 | 2.39 | 0.86 | 0.76 | 0.87 |
| **CC12.1** | 1.51 | 4.47 | 6.49 | 0.81 | 1.26 | 0.80 | 0.84 | 2.17 | 1.01 | 3.09 | 0.93 | 0.84 | 0.87 | 0.97 | 0.97 | 2.59 | 0.71 | 3.11 |
| **CV30** | 0.80 | 2.19 | 3.64 | 3.19 | 1.04 | 0.83 | 0.81 | 2.01 | 0.84 | 3.30 | 0.83 | 0.86 | 0.87 | 0.84 | 0.73 | 1.03 | 0.79 | 1.99 |
| **CB6** | 0.83 | 0.74 | 4.06 | 0.81 | 3.47 | 1.07 | 1.23 | 0.84 | 0.87 | 4.40 | 1.03 | 0.87 | 0.80 | 0.80 | 0.80 | 3.91 | 0.86 | 3.30 |
| **2-4** | 0.89 | 0.86 | 0.77 | 0.76 | 1.19 | 5.81 | 2.71 | 0.99 | 0.86 | 0.80 | 0.81 | 0.94 | 0.76 | 0.67 | 0.87 | 0.87 | 0.86 | 0.86 |
| **P2B-2F6** | 0.93 | 0.87 | 1.27 | 0.83 | 0.86 | 5.40 | 2.99 | 1.09 | 1.33 | 1.00 | 0.87 | 1.67 | 2.36 | 0.96 | 0.90 | 1.03 | 0.86 | 1.24 |
| **CC12.3** | 0.81 | 0.79 | 0.00 | 0.90 | 0.79 | 0.87 | 1.01 | 3.76 | 0.00 | 0.91 | 0.90 | 1.13 | 0.83 | 0.83 | 0.83 | 0.84 | 0.74 | 0.87 |
| **BD23** | 1.26 | 0.79 | 0.84 | 0.60 | 0.57 | 0.77 | 0.74 | 0.66 | 0.81 | 0.81 | 0.74 | 0.86 | 0.74 | 0.64 | 1.11 | 0.71 | 0.77 | 1.26 |
| **B38** | 0.81 | 1.11 | 2.19 | 0.74 | 0.03 | 1.24 | 1.17 | 1.13 | 0.94 | 2.06 | 1.26 | 0.87 | 0.84 | 1.20 | 0.94 | 1.00 | 1.11 | 1.06 |
| **REGN10987** | 0.96 | 0.80 | 0.83 | 0.60 | 0.69 | 0.84 | 0.80 | 0.59 | 0.76 | 0.83 | 3.84 | 0.83 | 0.79 | 0.64 | 0.83 | 0.74 | 0.76 | 0.87 |
| **C105** | 0.96 | 0.87 | 0.87 | 0.81 | 0.79 | 0.99 | 0.86 | 0.96 | 1.00 | 0.84 | 0.81 | 3.00 | 0.00 | 0.81 | 0.80 | 0.80 | 0.90 | 1.14 |
| **REGN10933** | 1.07 | 0.67 | 0.76 | 0.79 | 0.56 | 0.91 | 1.04 | 0.56 | 0.71 | 1.00 | 0.87 | 0.79 | 3.00 | 0.54 | 0.74 | 0.60 | 0.64 | 1.26 |
| **CR3022** | 0.73 | 0.79 | 0.84 | 0.77 | 0.76 | 0.77 | 0.81 | 0.76 | 0.79 | 0.79 | 0.77 | 0.94 | 0.73 | 3.16 | 0.74 | 0.76 | 0.80 | 1.13 |
| **LCA60** | 0.87 | 0.87 | 0.96 | 0.81 | 0.97 | 1.63 | 0.79 | 0.84 | 0.84 | 0.84 | 0.94 | 0.83 | 1.01 | 0.90 | 1.01 | 0.87 | 0.93 | 1.16 |
| **EY6A** | 0.97 | 0.81 | 0.66 | 0.64 | 0.77 | 0.83 | 0.81 | 0.61 | 0.79 | 0.86 | 0.87 | 0.79 | 0.73 | 0.71 | 0.84 | 2.06 | 0.80 | 1.10 |
| **4A8** | 1.17 | 0.87 | 0.83 | 0.69 | 0.79 | 0.79 | 0.81 | 0.74 | 0.76 | 0.76 | 0.80 | 0.80 | 0.76 | 0.80 | 0.77 | 1.77 | 0.83 | 0.77 |
| **XG83** | 0.74 | 0.86 | 0.81 | 0.90 | 0.83 | 0.84 | 1.60 | 0.77 | 0.81 | 0.81 | 0.87 | 0.77 | 0.81 | 1.93 | 0.79 | 0.79 | 0.79 | 4.19 |

**Table S3. Primer Sequences. (Reference 21).**

|  | **5'-3' sequence** |
| --- | --- |
| 5' L-VH 1 | ACAGGTGCCCACTCCCAGGTGCAG |
| 5' L-VH 3 | AAGGTGTCCAGTGTGARGTGCAG |
| 5' L-VH 4/6 | CCCAGATGGGTCCTGTCCCAGGTGCAG |
| 5' L-VH 5 | CAAGGAGTCTGTTCCGAGGTGCAG |
| HuIgG-const-anti | TCTTGTCCACCTTGGTGTTGCT |
| 5' AgeI VH1/5/7 | CTGCAACCGGTGTACATTCCGAGGTGCAGCTGGTGCAG |
| 5' AgeI VH3 | CTGCAACCGGTGTACATTCTGAGGTGCAGCTGGTGGAG |
| 5' AgeI VH3–23 | CTGCAACCGGTGTACATTCTGAGGTGCAGCTGTTGGAG |
| 5' AgeI VH4 | CTGCAACCGGTGTACATTCCCAGGTGCAGCTGCAGGAG |
| 5' AgeI VH 4–34 | CTGCAACCGGTGTACATTCCCAGGTGCAGCTACAGCAGTG |
| 5' AgeI VH 1–18 | CTGCAACCGGTGTACATTCCCAGGTTCAGCTGGTGCAG |
| 5' AgeI VH 1–24 | CTGCAACCGGTGTACATTCCCAGGTCCAGCTGGTACAG |
| 5' AgeI VH 3–9/30/33 | CTGCAACCGGTGTACATTCTGAAGTGCAGCTGGTGGAG |
| 5' AgeI VH 6–1 | CTGCAACCGGTGTACATTCCCAGGTACAGCTGCAGCAG |
| 3' SalI JH 1/2/4/5 | TGCGAAGTCGACGCTGAGGAGACGGTGACCAG |
| 3' SalI JH 3 | TGCGAAGTCGACGCTGAAGAGACGGTGACCATTG |
| 3' SalI JH 6 | TGCGAAGTCGACGCTGAGGAGACGGTGACCGTG |

|  | **5'-3' sequence** |
| --- | --- |
| 5' L Vk 1/2 | ATGAGGSTCCCYGCTCAGCTGCTGG |
| 5' L Vk 3 | CTCTTCCTCCTGCTACTCTGGCTCCCAG |
| 5' L Vk 4 | ATTTCTCTGTTGCTCTGGATCTCTG |
| 3' Ck 543–566 | GTTTCTCGTAGTCTGCTTTGCTCA |
| 5' Pan Vk | ATGACCCAGWCTCCABYCWCCCTG |
| 3' Ck 494–516 | GTGCTGTCCTTGCTGTCCTGCT |

|  | **5'-3' sequence** |
| --- | --- |
| 5' L Vλ 1 | GGTCCTGGGCCCAGTCTGTGCTG |
| 5' L Vλ 2 | GGTCCTGGGCCCAGTCTGCCCTG |
| 5' L Vλ 3 | GCTCTGTGACCTCCTATGAGCTG |
| 5' L Vλ 4/5 | GGTCTCTCTCSCAGCYTGTGCTG |
| 5' L Vλ 6 | GTTCTTGGGCCAATTTTATGCTG |
| 5' L Vλ 7 | GGTCCAATTCYCAGGCTGTGGTG |
| 5' L Vλ 8 | GAGTGGATTCTCAGACTGTGGTG |
| 3' Cλ | CACCAGTGTGGCCTTGTTGGCTTG |
| 5' AgeI Vλ 1 | CTGCTACCGGTTCCTGGGCCCAGTCTGTGCTGACKCAG |
| 5' AgeI Vλ 2 | CTGCTACCGGTTCCTGGGCCCAGTCTGCCCTGACTCAG |
| 5' AgeI Vλ 3 | CTGCTACCGGTTCTGTGACCTCCTATGAGCTGACWCAG |
| 5' AgeI Vλ 4/5 | CTGCTACCGGTTCTCTCTCSCAGCYTGTGCTGACTCA |
| 5' AgeI Vλ 6 | CTGCTACCGGTTCTTGGGCCAATTTTATGCTGACTCAG |
| 5' AgeI Vλ 7/8 | CTGCTACCGGTTCCAATTCYCAGRCTGTGGTGACYCAG |
| 3' XhoI Cλ | CTCCTCACTCGAGGGYGGGAACAGAGTG |
